# LGB Tobacco Control: Do Health Belief Model Constructs Predict Tobacco Use Intentions Differently between LGB and Heterosexual Individuals?

**DOI:** 10.3390/ijerph18137008

**Published:** 2021-06-30

**Authors:** Yachao Li, Bo Yang, Bryan Chen

**Affiliations:** 1 Department of Communication Studies, The College of New Jersey, Ewing, NJ 08628, USA; 2 Department of Public Health, The College of New Jersey, Ewing, NJ 08628, USA; chenb6@tcnj.edu; 3 Department of Communication, University of Arizona, Tucson, AZ 85721, USA; byang1@arizona.edu

**Keywords:** Health Belief Model, LGB, tobacco control, e-cigarettes, cigarettes

## Abstract

This research includes two studies testing whether the Health Belief Model (HBM) constructs predict tobacco use intentions differently between heterosexual and lesbian, gay, and bisexual (LGB) people. Focusing on cigarette smoking, Study 1 (*n* = 1808 U.S. adult current smokers) found that the perceived health threat and perceived benefits of smoking differently predicted intentions to continue smoking between heterosexual and LGB smokers. The perceived health threat of smoking had a weaker negative relationship and perceived benefits of smoking had a stronger positive relationship with smoking intentions among LGB smokers than heterosexual smokers. Focusing on vaping, Study 2 (*n* = 2801 U.S. adults) found that the perceived health threat and perceived barriers of vaping differentially predicted vaping intentions between heterosexual and LGB individuals. The perceived health threat of vaping only negatively predicted vaping intentions among heterosexual people. Perceived barriers to vaping had a stronger negative relationship with intentions to vape among LGB people than among heterosexual people. Our finding suggests that compared to perceptions of tobacco-related health consequences (perceived heath threat), behavioral perceptions (perceived benefits and barriers) may have stronger impacts on tobacco use intentions among LGB people. Thus, efforts focusing on reducing tobacco-related disparities among the LGB community should address perceived benefits and barriers of tobacco use.

## 1. Introduction

Tobacco-related health disparities are growing among the lesbian, gay, and bisexual (LGB) community. Compared with heterosexual people, LGB individuals have reported disproportionately high rates of tobacco use, including both traditional cigarettes and electronic cigarettes (e-cigarettes). According to the 2016 U.S. National Health Interview Survey, 21% of LGB adults smoked cigarettes, compared to 15% of heterosexual adults [1]; 19% of LGB adults aged between 18 and 24 indicated that they were currently using cigarettes, compared to 12% of heterosexual young adults [2]. In addition, 1 in 3 sexual minority individuals used e-cigarettes at least once, compared to that 1 in 5 heterosexual people who have ever vaped [3]. Tobacco use has direct adverse health consequences [4,5] and may aggravate common health issues faced by the LGB community, such as cancer [6], cardiovascular diseases [7], and HIV and sexually transmitted diseases [8]. Additionally, tobacco use carries significant social and economic burdens [9]. Thus, reducing tobacco use and related health disparities among the LGB community is crucial.

Despite the significance and urgency of reducing tobacco use among LGB people, there have been few LGB-targeted anti-tobacco campaigns in the U.S. [10]. In addition, few studies have attempted to understand tobacco use interventions among LGB people [11]. Indeed, among 144 articles published between 2004 and 2019 that examined anti-tobacco campaigns aimed at vulnerable populations, only 2% of the studies focused on sexual minority populations [12]. Thus, much remains to be known about how to develop more effective tobacco interventions for LGB people. Our study aimed to provide some insights into this topic. We utilized the Health Belief Mode (HBM) to explore factors predicting LGB people’s tobacco use intentions. Furthermore, we examined whether HBM constructs predict tobacco use differently between LGB and heterosexual people. As a result, we can inform tobacco control interventions aiming to reduce tobacco-related health disparities among the LGB community.

As one of the most widely applied health behavior theories [13], HBM identifies multiple cognitive antecedents to health-related behavior [14] and is very helpful for designing effective public health interventions. Specifically, HBM states that three factors determine people’s likelihood of engaging in a behavior, including (a) perceived threat, consisting of perceived susceptibility and perceived severity of a health condition and its consequences, (b) perceived benefits of engaging a behavior, and (c) perceived barriers to performing a behavior. When people perceive that they are facing a high health threat and consider that the benefits of engaging in a health-promoting behavior outweigh the barriers to performing the behavior, they will likely perform the behavior.

Several studies have shown that HBM is a useful model to understand tobacco use and cessation behaviors. For instance, a greater perceived threat of smoking-related diseases, higher perceived benefits of quitting, lower perceived barriers to quitting, and higher self-efficacy for quitting predicted a greater likelihood of quitting smoking [15,16]. In a longitudinal study, scholars found that a lower perceived threat of smoking predicted e-cigarette use in non-smokers [17]. Another study found that higher perceived benefits of e-cigarette use were associated with having tried e-cigarettes among both smokers and non-smokers [18]. Given these studies, HBM might be helpful for understanding LGB people’s tobacco use behaviors. 

Emerging evidence has shown that LGB and heterosexual people could have different tobacco use motivations, attitudes, and behaviors. In a heteronormative society that assumes and favors heterosexuality, LGB individuals need to cope with a variety of forms of minority stress, such as concealment of their sexual orientation as well as societal stigma and discrimination [19]. As such, LGB people might be more likely than their heterosexual counterparts to use tobacco products for stress relief [20]. Another study found that LGB smokers were also less likely to call tobacco quit lines than their heterosexual counterparts, partly due to their lack of knowledge of what quit lines offer [21]. With these differences between LGB and heterosexual people, it is useful to explore whether the predictive power of HBM differs between LGB and heterosexual individuals. In this project, we conducted secondary data analyses of two larger studies of people’s beliefs, behaviors, and risk communication concerning cigarettes and e-cigarettes. We examined how HBM constructs predicted intentions to continue smoking (Study 1) and intentions to vape (Study 2) between heterosexual and LGB individuals.

## 2. Materials and Methods

### 2.1. Participants

Data were from two larger studies focusing on tobacco product use, beliefs, and communication. The two datasets included different HBM variables. One focused on cigarette smoking, and the other focused on vaping. Thus, we analyzed datasets of the two studies separately and reported their method and results as Study 1 (cigarette smoking, e.g., [22,23]) and Study 2 (vaping, e.g., [24]). Study 1 recruited 1906 U.S. adult current smokers (i.e., those who had used 100 cigarettes in their life and were currently smoking some days or every day) and recent former smokers (smokers who quit in the past two years). For the purpose of this study, recent former smokers were not included because they did not report their intentions to continue smoking. Due to missing data, the final sample included in data analyses were 1808 smokers. Study 2 included 2801 U.S. adults with various tobacco use experiences. In both studies, participants were recruited via multiple online recruitment strategies (e.g., web banners, affiliate marketing, pay-per-click) by a market research company, Toluna. Table 1 summarizes the demographic characteristics of participants.

### 2.2. Procedure

The two larger studies were conducted on Toluna’s website. In Study 1, participants first provided informed consent, and reported their sociodemographic information and tobacco use experience. Participants then saw some tobacco risk messages. After message exposure, they responded to questions assessing the HBM constructs related to smoking (see Section 2.3 Study 1 Measures). The effects of the messages have been explored in our prior publications and were not the focus of the present study [22,23]. To remove the effects of the messages, we controlled for the message factor in our regression analysis exploring the relationship between HBM constructs and intentions to continue smoking. Upon completion of the study, all participants were debriefed and directed to smoking cessation resources. The study was conducted in accordance with the Declaration of Helsinki, and its protocol was approved by Georgia State University’s Institutional Review Board (IRB).

Study 2 had a similar procedure to Study 1. After providing informed consent, participants reported their sociodemographic information and tobacco use. They then saw some e-cigarette risk messages which might evoke different levels of various e-cigarette related beliefs. After message exposure, participants responded to questions assessing the HBM constructs related to vaping (see Section 2.4). The effects of the messages were addressed elsewhere [24] and were not the interest of the present research. To remove the effects of the messages, we controlled for the message factor in our data analyses. Upon completion of the study, all participants were debriefed and directed to smoking cessation resources. The study was conducted in accordance with the Declaration of Helsinki, and its protocol was approved by Georgia State University IRB.

### 2.3. Study 1 Measures

Table 2 summarizes variable means and standard deviations overall and by sexual orientation.

#### 2.3.1. Perceived Health Threat of Smoking

On a 9-point Likert-type scale (1 = not at all, 9 = extremely), participants responded to 2 questions: “If you develop a smoking-related disease, how severe or serious will it be?” (perceived severity) and “How likely is it for you to develop a smoking-related disease?” (perceived susceptibility) [25]. The two items were averaged to index the perceived health threat of smoking (*r* = 0.76, *p* < 0.001).

#### 2.3.2. Perceived Benefits of Smoking

Participants reported on a 7-point scale (0 = no chance, 6 = very good chance) to indicate how likely they thought each of 4 positive outcomes (“Look cool,” “Feel more relaxed,” “Have better concentration,” and “Be more popular”) would happen if they started and continued to smoke every day [26]. The 4 items were averaged to assess perceive benefits of smoking (α = 0.82).

#### 2.3.3. Perceived Barriers to Smoking

On a 7-point scale (0 = no chance, 6 = very good chance), respondents reported the likelihood of having each of 5 negative consequences (e.g., “Become addicted” “Early/premature death”) if they started and continued to smoke every day [26]. The 5 items were averaged to evaluate perceived barriers to smoking (α = 0.91).

#### 2.3.4. Intentions to Continue Smoking

On a 4-point scale (1 = definitely will not, 4 = definitely will), respondents answered how much they intended to “reduce the number of cigarettes” consumed in a day. Responses were reverse coded to assess intentions to continue smoking.

#### 2.3.5. Covariates

Control variables included age, gender (0 = cisgender and transgender man, 1 = cisgender and transgender woman), race/ethnicity (0 = White, 1 = people of color), education (0 = below college, 1 = college and above), nicotine dependence (Heaviness of Smoking Index, 0–6 scale [27,28]), current e-cigarette use (0 = no, 1 = yes), and messages.

### 2.4. Study 2 Measures

Table 2 summarizes variable means and standard deviations overall and by sexual orientation.

#### 2.4.1. The Perceived Health Threat of Vaping

On a 9-point Likert-type scale (1 = not at all, 9 = extremely), participants responded to one question assessing perceived severity (“If you develop a disease from using e-cigarettes, how severe or serious will it be?”) and 2 items evaluating perceived susceptibility (“I am at risk of developing cancer from using e-cigarettes,” “It is likely that my health will suffer from using e-cigarettes.”) [25]. The 3 items were averaged to index the perceived health threat of vaping (α = 0.81).

#### 2.4.2. Perceived Benefits of Vaping

Participants reported on a 7-point scale (0 = no chance, 6 = very good chance) to indicate how likely they thought each of 4 positive outcomes (“Look cool,” “Feel more relaxed,” “Have better concentration,” and “Be more popular”) would be to happen if they started and continued to vape every day [26]. The 4 items were averaged to index perceived benefits of vaping (α = 0.86).

#### 2.4.3. Perceived Barriers to Vaping

On a 7-point scale (0 = no chance, 6 = very good chance), respondents reported on the likelihood of having each of 5 negative consequences (e.g., “Become addicted” “Early/premature death”) if they started and continued to vape every day [26]. The 5 items were averaged to assess perceived barriers to vaping (α = 0.86).

#### 2.4.4. Intentions to Vape

Participants indicated how open they were to trying e-cigarettes in the future (single item) on a 9-point scale (1 = not at all open, 9 = extremely open) [29].

#### 2.4.5. Covariates

Study 2 had the same control variables as study 1, which included age, gender (0 = cisgender and transgender man, 1 = cisgender and transgender woman), race/ethnicity (0 = White, 1 = people of color), education (0 = below college, 1 = college and above), nicotine dependence (Heaviness of Smoking Index, 0–6 scale [27,28]), current e-cigarette use (0 = no, 1 = yes), and messages. We also controlled for current cigarette use (0 = no, 1 = yes) when analyzing Study 2 data.

### 2.5. Data Analysis

Hierarchical regression analyses tested if and how sexual orientation moderated the relationship between HBM constructs and tobacco use intentions. When analyzing Study 1 data, the regression model predicting intentions to continue smoking included three blocks of variables that were entered in three consecutive steps. Block 1 entered in step 1 had all the control variables detailed in Section 2.3, including gender, race/ethnicity, education, nicotine dependence, current e-cigarette use, and messages. Block 2 included LGB identity (0 = Heterosexual, 1 = LGB) and HBM variables (The perceived health threat of smoking, perceived benefits of smoking, and perceived barriers to smoking). Block 3 included interactions between LGB and each HBM construct from Block 2. Significant interactions in Block 3 were further explored through Hayes’ PROCESS V3.5.3 macro [30] Model 1. Specifically, for significant interactions, we plotted the relationship between an HBM construct and intentions to continue smoking in LGB and heterosexual individuals, respectively. We followed the same analytic approach to analyze Study 2 data: a regression model predicting intentions to vape that included three blocks of variables. Block 1 had all the control variables detailed in Section 2.4. Block 2 included LGB identity (0 = Heterosexual, 1 = LGB) and HBM variables (the perceived health threat of smoking, perceived benefits of smoking, and perceived barriers to smoking). Block 3 included the interaction terms between LGB and each HBM construct from Block 2. Hayes’ PROCESS V3.5.3 macro [30] Model 1 explored any significant interactions predicting intentions to vape in Block 3. We performed all analyses in SPSS V.27 with the significance level set at *p* < 0.05.

## 3. Results

### 3.1. Study 1 Results

Table 3 summarizes the results. After controlling for participants’ age, gender, race/ethnicity, education, current e-cigarette use, nicotine dependence, and messages, the perceived health threat of smoking (β = −0.33, *p* < 0.001) and perceived barriers to smoking (β = −0.09, *p* < 0.001) negatively predicted intentions to continue smoking. There was a positive relationship between perceived benefits of smoking and intentions to continue smoking (β = 0.06, *p* = 0.004). In addition, the interaction between LGB and the perceived health threat of smoking positively predicted intentions to continue smoking (β = 0.25, *p* < 0.001). The interaction between LGB and perceived benefits of smoking also positively predicted intentions to continue smoking (β = 0.10, *p* = 0.013). Thus, LGB significantly moderated the relationships between the perceived health threat and intentions to continue smoking and between perceived benefits of smoking and intentions to continue smoking.

**Figure 1:**
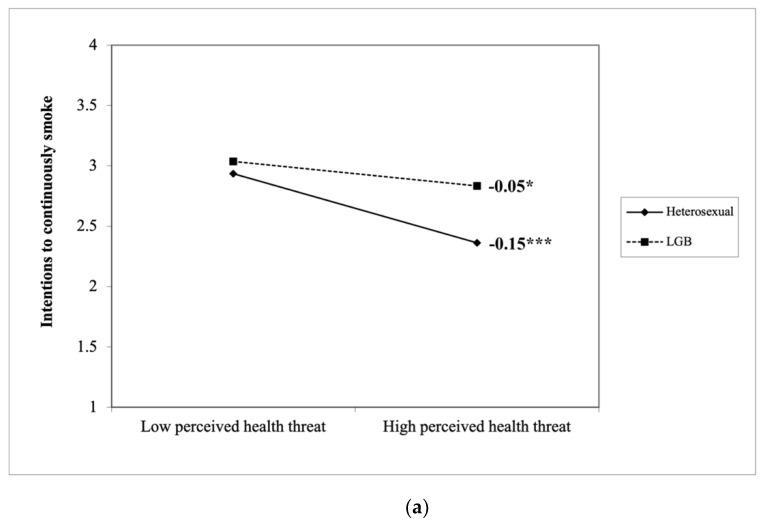

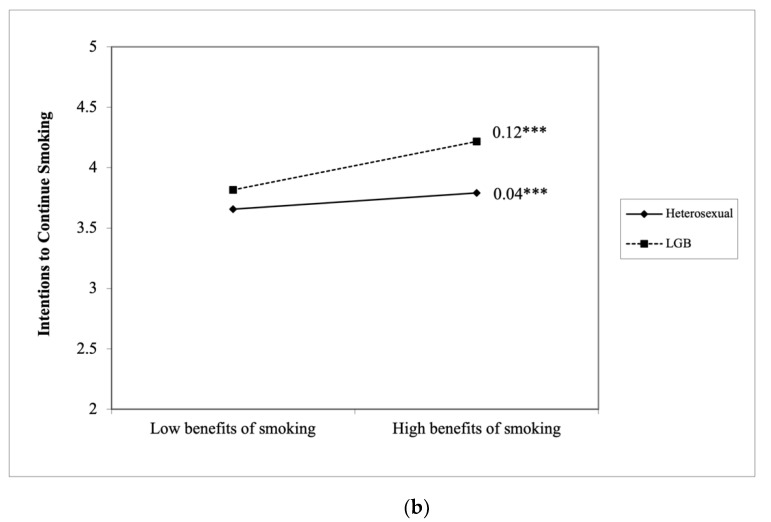
(**a**) LGB moderates how the perceived health threat of smoking predicts intentions to continue smoking. (**b**) LGB moderates how perceived benefits of smoking predicts intentions to continue smoking. *** *p* < 0.001, * *p* < 0.05.

Specifically, simple slopes analyses showed that the perceived heath threat of smoking negatively predicted intentions to continue smoking among both LGB and heterosexual smokers (B_LGB_ = −0.05, *SE*_LGB_ = 0.03, *p* = 0.039 vs. B_heterosexual_ = −0.15, *SE*_heterosexual_ = 0.01, *p* < 0.001). Compared to heterosexual smokers, the perceived heath threat of smoking had a weaker negative relationship with intentions to continue smoking among LGB smokers, *t*(1806) = 7.24, *p* < 0.001 (see Figure 1a). Perceived benefits of smoking positively predicted intentions to continue smoking among both groups (B_LGB_ = 0.12, *SE*_LGB_ = 0.03, *p* < 0.001 vs. B_heterosexual_ = 0.04, *SE*_heterosexual_ = 0.01, *p* < 0.001). Perceived benefits of smoking had a stronger positive relationship with intentions to continue smoking among LGB smokers than heterosexual smokers, *t*(1806) = 6.59, *p* < 0.001 (see Figure 1b).

### 3.2. Study 2 Results

Results for vaping (Table 4) shows that after controlling for participants’ age, gender, race/ethnicity, education, current cigarette use, current e-cigarette use, nicotine dependence, and messages, perceived benefits of vaping positively predicted intentions to vape (β = 0.27, *p* < 0.001). Perceived barriers to vaping negatively predicted intentions to vape (β = −0.28, *p* < 0.001). Yet, the perceived health threat of vaping did not significantly predict vape intentions (β = −0.01, *p* = 0.337). Moreover, the interaction between LGB and The perceived health threat of vaping positively predicted intentions to vape (β = 0.08, *p* = 0.027). The interaction between LGB and perceived barriers to vaping negatively predicted intentions to vape (β = −0.10, *p* = 0.005). Thus, LGB significantly moderated the relationships between the perceived health threat and intentions to vape, and between perceived barriers to vaping and intentions to vape.

**Figure 2:**
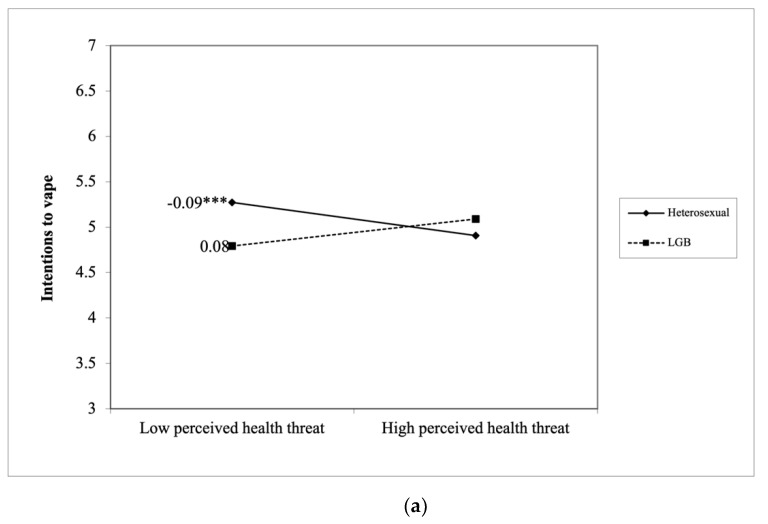

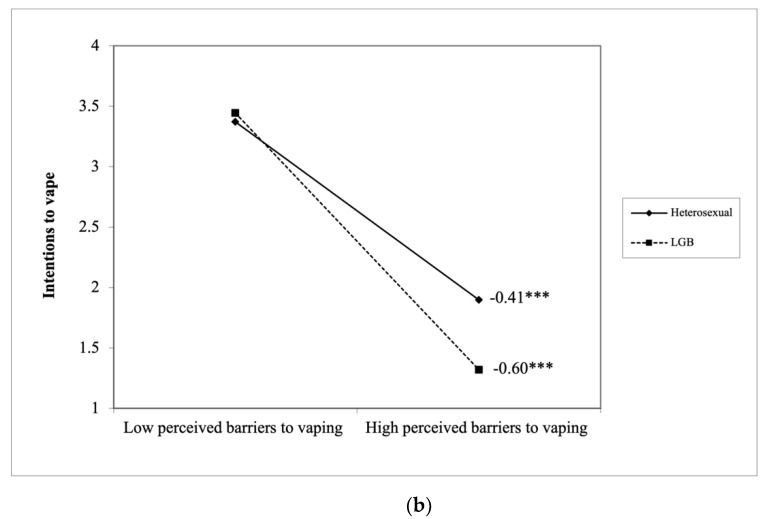
(**a**) LGB moderates how the perceived health threat of vaping predicts intentions to vape. (**b**) LGB moderates how perceived barriers to vaping predicts intentions to vape. *** *p* < 0.001.

Specifically, among LGB people, the perceived heath threat of vaping did not predict intentions to vape (B_LGB_ = 0.08, *SE*_LGB_ = 0.05, *p* = 0.181), whereas among heterosexual participants, perceived heath threat of vaping negatively predicted intentions to vape (B_heterosexual_ = −0.09, *SE*_heterosexual_ = 0.02, *p* < 0.001), *t*(2799) = 6.95, *p* < 0.001 (see Figure 2a). In addition, perceived barriers to vaping negatively predicted intentions to vape among both LGB and heterosexual participants (B_LGB_ = −0.60, *SE*_LGB_ = 0.06, *p* < 0.001 vs. B_heterosexual_ = −0.41, *SE*_heterosexual_ = 0.03, *p* < 0.001). Compared to heterosexual participants, perceived barriers to vaping had a stronger negative relationship with intentions to vape among LGB people, *t*(2799) = −5.59, *p* < 0.001 (see Figure 2b). 

## 4. Discussion

The LGB community has been experiencing heightened tobacco-related health disparities. However, little is known about the most important factors to target in LGB people to eliminate the disparities. To address the research gap, this study explored whether HBM factors (the perceived health threat, perceived benefits, and perceive barriers) may predict tobacco use intentions differently between LGB and heterosexual people. 

In the contexts of traditional and electronic cigarette use, perceived benefits and barriers of tobacco use seemed to be more important for LGB people’s tobacco use intentions. Since the early 1990s, the LGB population has been a focus of the tobacco industry [31]. Nowadays, the tobacco industry openly targets LGB people, including sexual minority youth, by placing ads in LGB media and bars, by sponsoring Pride events and LGB organizations, and by highlighting cherished LGBT values, such as freedom, choice, and pride, to make tobacco use more appealing to the community [8,32,33]. The aggressive LGB-targeted marketing may mislead LGB people into believing that smoking and vaping are inextricably linked to their LGB identity, which may increase the salience and thus importance of benefits and barriers to tobacco use in predicting LGB people’s tobacco use.

Our study also found that for both cigarette smoking and vaping, the perceived health threat of these behaviors mattered less to LGB people than to their heterosexual counterparts. The results were consistent with prior research. Lee and colleagues found that LGB smokers had less favorable attitudes toward warning labels that focused on the health consequences of tobacco use [34]. One possible explanation for the weaker role of the perceived health threat in predicting tobacco use intentions among LGB people might be that tobacco use is central to LGB identity so that LGB people might overlook health risks of tobacco use. Moreover, the needs to relieve minority stress commonly experienced by LGB people may override their concerns about health consequences of tobacco use.

Notably, many tobacco education campaigns nowadays use messages to increase people’s the perceived health threat of using tobacco products. Based on our finding, however, such messaging might not work effectively in LGB people. As such, our findings raised an important issue for tobacco control research and efforts. As discussed earlier, very few studies have thoroughly evaluated LGB people’s tobacco use motivations, attitudes, and behaviors and how to most effectively encourage these people to stay away from tobacco products. Our study did find some nuances existing in the tobacco-related beliefs among LGB and heterosexual people. Following our study, more research should be conducted to better understand how to better promote tobacco product prevention and cessation among LGB people. 

Our results have practical implications for designing anti-tobacco messages to address tobacco-related health disparities among LGB people. Given the limited impacts of the perceived health threat on sexual minority people’s tobacco use intentions, LGB-targeted anti-tobacco messages should not just focus on the health consequences of tobacco use. Instead, because perceived benefits and barriers of tobacco use appear to have stronger influences on LGB people’s tobacco use intentions, messages should aim to reduce LGB people’s perceived benefits of tobacco use and increase their perceived barriers. As we found some differences between LGB and heterosexual individuals’ tobacco-related beliefs, it is strongly suggested that future tobacco education messages should be adequately pretested and assessed among sexual minority individuals with LGB people’s needs better understood.

There are several limitations of this study. First, this study combined lesbian, gay, and bisexual participants into one group, and compared it with heterosexual participants. We did this because of the relatively small sample size of each subgroup, and that variable means did not differ between the subgroups. However, research has identified differences in tobacco message processing between sexual and gender minority subgroups [20]. Future studies should collect more diverse samples, including transgender participants, to explore if there are nuances between the sexual and gender minority subgroups in tobacco-related beliefs. Moreover, due to the nature of secondary data analyses, we were only able to examine three key HBM constructs (i.e., The perceived health threat, perceived benefits, and perceived barriers) and left out other HBM variables, such as self-efficacy and cues to action. In addition, this study examined specifically how HBM constructs were related to intentions to continue smoking and vaping. To better inform tobacco control interventions, future studies may want to explore the factors shaping LGB people’s tobacco use cessation behaviors. Finally, data were cross-sectional, which precluded claims of causality.

## 5. Conclusions

In conclusion, this study demonstrates that HBM constructs predicted tobacco use intentions differently between heterosexual and LGB individuals. In the contexts of both smoking and vaping, the perceived health threat seemed to have weaker predictive power in LGB vs. heterosexual people. In contrast, perceived benefits and barriers of tobacco use appeared to predict LGB people’s tobacco use intentions more strongly than those of straight people. The differences may be attributed to the increased minority stress facing LGB people as well as the prolonged, aggressive tobacco marketing targeting the LGB community. Tobacco education messages aiming to eliminate tobacco-related health disparities among the LGB community may want to target LGB people’s perceived benefits and barriers of tobacco use.

## Figures and Tables

**Table 1:** Sample Characteristics.

Characteristics	Study 1 (*n* = 1808, %)	Study 2 (*n* = 2801, %)
Age (years)		
18–29	23.00	19.35
30–44	33.10	24.21
45–59	23.90	25.35
60+	20.00	31.10
Gender		
Woman	54.32	50.07
Man	44.97	48.98
Transgender	0.72	0.32
Sexual orientation		
Heterosexual	88.99	90.54
Lesbian or gay	4.04	3.53
Bisexual	6.08	4.82
Queer or other	0.88	1.11
Race/Ethnicity		
White	72.84	78.89
People of color	27.16	21.11
Education		
College or above	37.44	34.99
Some college or below	62.56	65.01
Current e-cigarette use		
Yes	53.76	76.12
No	46.24	23.88
Current cigarette use		
Yes	100.00	41.31
No	0.00	48.69

**Table 2:** Variable Means and Standard Deviations.

Variable	Heterosexual			LGB			Full Sample		
	Mean	Median	*SD*	Mean	Median	*SD*	Mean	Median	*SD*
Study 1 (*n* = 1808)									
Perceived health threat of smoking	6.46	6.50	1.86	6.31	6.50	1.94	6.44	6.50	1.87
Benefits of smoking	2.81	2.75	1.66	3.12	3.00	1.73	2.85	2.75	1.67
Barriers to smoking	4.75	5.20	1.36	4.69	5.00	1.39	4.74	5.20	1.37
Intentions to continue smoking	2.43	2.33	0.82	2.66	2.67	0.82	2.45	2.33	0.81
Study 2 (*n* = 2801)									
Perceived health threat of vaping	3.87	3.67	2.10	4.13	4.00	2.25	3.90	3.67	2.12
Benefits of vaping	2.43	2.00	2.26	3.07	2.75	2.28	2.49	2.25	2.27
Barriers to vaping	4.85	5.00	1.76	4.51	4.80	1.97	4.82	5.00	1.78
Intentions to vape	3.17	1.00	2.78	3.58	2.00	2.93	3.21	1.00	2.80

Note: Full sample included both heterosexual and LGB participants. The perceived health threat of smoking and the perceived health threat of vaping ranged from 1 to 9. Benefits of smoking, barriers to smoking, benefits of vaping, and barriers to vaping ranged from 0 to 6. Intentions to continue smoking ranged from 1 to 4. Intentions to vape ranged from 1 to 9. *SD* = Standard deviation.

**Table 3:** Hierarchical Regression Analyses Predicting Intentions to Continue Smoking among Smokers.

Variable	*B*	*SE*	β	*p*	95% CI for *B*		$∆R^{2}$
					*LL*	*UL*	
Block 1: Controls							0.091 ***
Block 2:							0.154 ***
LGB	0.28	0.05	0.11	<0.001	0.17	0.38	
Perceived health threat	−0.14	0.01	−0.33	<0.001	−0.16	−0.12	
Benefits of smoking	0.03	0.01	0.06	0.004	0.01	0.05	
Barriers to smoking	−0.05	0.01	−0.09	<0.001	−0.08	−0.03	
Block 3:							
3A. LGB × Threat	0.10	0.03	0.25	<0.001	0.04	0.15	0.005 ***
3B. LGB × Benefits	0.08	0.03	0.10	0.013	0.02	0.14	0.003 *
3C. LGB × Barriers	0.03	0.04	0.06	0.407	−0.04	0.11	0.000

Note. Age, gender, race/ethnicity, education, current e-cigarette use, nicotine dependence, and messages were controlled. Statistics for Block 2 and 3 variables were from the first time when the variables were added into the models. All equations in Block 3 (3A-3C) included variables in Blocks 1 and 2, in addition to one interaction term between LGB and one Health Belief Model construct. Thus, Block 3A included control variables, LGB (0 = no, 1 = yes), the perceived health threat of smoking, perceived benefits of smoking, perceived barriers to smoking, and the interaction between LGB and perceived heath threat of smoking (LGB × Threat). Block 3B included control variables, LGB, the perceived health threat of smoking, perceived benefits of smoking, perceived barriers to smoking, and the interaction between LGB and perceived benefits of smoking (LGB × Benefits). Block 3C included control variables, LGB, the perceived health threat of smoking, perceived benefits of smoking, perceived barriers to smoking, and the interaction between LGB and perceived barriers to smoking (LGB × Barriers). We tested each interaction item individually in the model to avoid multicollinear (all interactions included LGB). *B* = unstandardized coefficient, *SE* = standard error for the unstandardized coefficient, *β* = standardized coefficient, CI = confident interval, *LL* = lower limit, *UL* = upper limit. *** *p* < 0.001, * *p* < 0.05.

**Table 4:** Hierarchical Regression Analyses Predicting Intentions to Vape.

Variable	*B*	*SE*	β	*p*	95% CI for *B*		$∆R^{2}$
					*LL*	*UL*	
Block 1: Controls							0.396 ***
Block 2:							0.107 ***
LGB	−0.20	0.13	−0.02	0.114	−0.46	0.05	
Perceived health threat	−0.02	0.02	−0.01	0.337	−0.06	0.02	
Benefits of vaping	0.33	0.02	0.27	<0.001	0.30	0.37	
Barriers to vaping	−0.44	0.02	−0.28	<0.001	−0.48	−0.39	
Block 3:							
3A. LGB × Threat	0.16	0.07	0.08	0.021	0.02	0.29	0.001 *
3B. LGB × Benefits	−0.06	0.06	−0.02	0.314	−0.17	0.05	0.000
3C. LGB × Barriers	−0.18	0.07	−0.10	0.005	−0.31	−0.05	0.001 **

Note. Age, gender, race/ethnicity, education, current e-cigarette use, current cigarette use, nicotine dependence, and messages were controlled in all models. Statistics for Block 2 and 3 variables were from the first time when the variables were added into the models. All equations in Block 3 (3A-3C) included variables in Blocks 1 and 2, in addition to one interaction term between LGB and one Health Belief Model construct. Thus, Block 3A included control variables, LGB (0 = no, 1 = yes), the perceived health threat of vaping, perceived benefits of vaping, perceived barriers to vaping, and the interaction between LGB and perceived heath threat of vaping (LGB × Threat). Block 3B included control variables, LGB, the perceived health threat of vaping, perceived benefits of vaping, perceived barriers to vaping, and the interaction between LGB and perceived benefits of vaping (LGB × Benefits). Block 3C included control variables, LGB, the perceived health threat of vaping, perceived benefits of vaping, perceived barriers to vaping, and the interaction between LGB and perceived barriers to vaping (LGB × Barriers). We tested each interaction item individually in the model to avoid multicollinearity (all interactions included LGB). *B* = unstandardized coefficient, *SE* = standard error for the unstandardized coefficient, *β* = standardized coefficient, CI = confident interval, *LL* = lower limit, *UL* = upper limit. *** *p* < 0.001, ** *p* < 0.01, * *p* < 0.05.

## Data Availability

Data are available upon reasonable request.
